# Levetiracetam ameliorates epileptogenesis by modulating the adenosinergic pathway in a kindling model of epilepsy in mice

**DOI:** 10.55730/1300-0144.5669

**Published:** 2023-03-07

**Authors:** Muhammad JAMAL, Muhammad AZAM, Salman Ali KHAN, Zaheer UL-HAQ, Shabana Usman SIMJEE

**Affiliations:** 1 H.E.J. Research Institute of Chemistry, International Center for Chemical and Biological Sciences, Faculty of Sciences, University of Karachi, Karachi, Pakistan; 2 Dr. Panjwani Center for Molecular Medicine and Drug Research, International Center for Chemical and Biological Sciences, Faculty of Sciences, University of Karachi, Karachi, Pakistan

**Keywords:** Levetiracetam, adenosinergic pathway, seizure, kindling model, epilepsy

## Abstract

**Background:**

Levetiracetam (LEV) has been found to have an antihyperalgesic effect via acting on the adenosine system. However, the effects of LEV on the modulation of the adenosine system in the brain have not been elucidated in the prevention of seizures and epilepsy. The present study aimed to explore the possible LEV mechanisms of action in the adenosine signaling systems in an animal model of epilepsy.

**Methodology:**

A docking study was initially performed to determine the possible interaction of LEV with adenosine A1 receptors (A1Rs) and equilibrative nucleoside transporters-1 (ENT1). The experimental study was divided into an acute seizure test (32 mice distributed into 4 groups) and a chronic kindling model study (40 mice distributed into 5 groups), followed by gene expression analysis and immunohistochemistry. The kindling model lasted 26 days and took 13 subconvulsive doses of pentylenetetrazole (PTZ) to completely kindle the mice in the PTZ control group. Gene expression changes in the A1Rs, potassium inwardly-rectifying channel 3.2 (Kir3.2), and ENT1 in the brain tissue samples of the mice following treatment with LEV were analyzed using reverse transcription-quantitative polymerase chain reaction, and immunohistochemistry was performed for the A1R protein expression.

**Results:**

Docking studies predicted a significant interaction of LEV with A1Rs and ENT1 proteins. Results from the acute testing revealed that caffeine (100 mg/kg) and 8-cyclopentyl-1,3-dipropylxanthine (25 mg/kg) significantly reversed the antiseizure effects of LEV by reversing the percent protection and shortening the onset of the first myoclonic jerk (FMJ) and generalized clonic seizures (GCSs). In the PTZ-induced kindling, LEV demonstrated an increased gene expression of A1Rs and Kir3.2 in the brain. LEV also significantly reduced the gene expression of ENT1. Furthermore, the immunohistochemical analysis showed that LEV increased the protein expression of A1Rs in the brain.

**Conclusion:**

Based on these results, it can be concluded that LEV modulates epileptogenesis by acting on the adenosine pathway in the central nervous system.

## 1. Introduction

Epilepsy is a chronic, severe, and devastating neurological condition characterized by repeated intermittent seizures that affect over 65 million people worldwide [1, 2]. Focal seizures occur more frequently in both adults and children compared to generalized seizures. Focal seizures can also be worsened by other neurological conditions, such as stroke, tumors, and neurodegenerative disorders. According to reports, the first seizure occurs months to years after brain damage, and this time span is known as the latent phase [3]. Changes in the neuronal architecture during the latent period include gliosis, neurodegeneration, mossy fiber sprouting, release of neuroinflammatory mediators, and synaptic reconfiguration [4]. Based on these changes, epileptogenesis is the insult-induced transformation of a nonepileptic brain into an epileptic brain [5, 6]. Halting the injury-induced alterations and restricting their progression in the brain could provide an effective treatment for acquired epileptic conditions. [7].

The treatment of epilepsy with currently available antiepileptic drugs (AEDs) has an extensive side effect profile that has contributed to patients’ noncompliance with medication. Novel medications with fewer side effects and a larger spectrum of efficacy in epileptic patients have been developed. Among these newer drugs, levetiracetam (LEV) has been identified as the safest AED in terms of an adverse effect profile [8]. It has been utilized in the treatment of epilepsy as a monotherapy [9, 10], adjuvant therapy, and prophylactically to prevent posttraumatic seizures [11]. Recently, LEV has effectively been used for treating neonatal seizures [12], neuropathic pain, and hyperalgesia [13]. It was reported that the antihyperalgesic effects of LEV are related to its activity on A1 receptors (A1Rs), and that pharmacological blockage of A1Rs in the periphery by caffeine or 8-cyclopentyl-1,3-dipropylxanthine (DPCPX) reduced the benefits of this drug [14]. However, the antiepileptic effects of LEV through the adenosine mechanism has not previously been observed. Therefore, the current research focused on exploring the effect of LEV on the central nervous system adenosinergic pathway.

Adenosine is an endogenous anticonvulsant chemical in the brain that either directly regulates neuron hyperexcitation through its receptors or indirectly with the modulation of the release of other neurotransmitters [15, 16]. There are 4 subtypes of adenosine receptors, known as A1, A2A, A2B, and A3, which are distributed throughout the body and serve a variety of physiological functions. The antiepileptic effect of adenosine has been linked to its action on inhibitory A1Rs. The activation of A1Rs has an anticonvulsant effect and may help to reduce the frequency and severity of seizures [17–19]. Research has shown that there is a functional interaction between potassium inwardly-rectifying channel 3.2 (Kir3.2) and A1Rs. The activation of A1Rs has been demonstrated to increase the activity of Kir3.2, increasing potassium conductance and hyperpolarization of the cell membrane. This function has been hypothesized to contribute to adenosine’s anticonvulsant and neuroprotective properties, as it is known to lower neuron excitability and protect against seizures [20, 21].

Similarly, the level of adenosine in the brain is regulated by various transporters. Among these, equilibrative nucleoside transporter-1 (ENT1) is associated with the epileptic disorder. ENT1 is a transporter that helps to reuptake adenosine into neurons. Studies have shown that the changes in the expression of ENT1 are associated with epilepsy. Decreased ENT1 expression may lead to increased adenosine levels in the brain, thus reducing the likelihood of seizures. Conversely, an increased ENT1 expression may lead to decreased adenosine levels and an increased risk of seizures [20].

Since the antihyperalgesic activity of LEV in the periphery was evaluated via the A1Rs, the antiepileptic effects of LEV were analyzed herein via the modulation of an adenosinergic pathway in acute and kindling models of epilepsy.

## 2. Materials and methods

### 2.1. Study design

The experimental study was divided into 2 phases along with a computational study, i.e. docking studies, acute seizure tests, and a chronic kindling model study, followed by gene expression analysis and immunohistochemistry. A total of 32 mice (n = 8 mice/group) were distributed into 4 groups for the acute seizure tests, while 40 mice (n = 8 mice/group) were used for the kindling model of epileptogenesis. The kindling study lasted 26 days, with the administration of 13 subconvulsive doses of pentylenetetrazole (PTZ) to completely kindle the mice in the PTZ control group.

### 2.2. Chemicals

The PTZ, caffeine, DPCPX, and diazepam were purchased from Sigma-Aldrich (Gillingham, Dorset, UK), the LEV was purchased from Helix Pharmaceutical (Karachi City, Sindh, Pakistan). The drugs were dissolved in distilled water, except for the DPCPX, which was diluted in 5% dimethyl sulfoxide (DMSO). The injection volume was approximately 10 mL/kg of the body weight. The primers and their sequences were purchased from Penicon Ltd. (Karachi, Pakistan) and are provided in Table 1. The antibodies used in the immunohistochemistry were purchased from Cloud Clone Corp. (Houston, TX, USA).

### 2.3. The mice

Male Naval Medical Research Institute (NMRI) mice weighing 18–22 g, which had access to food and water ad libitum were used. The experiments were conducted in accordance with the institutional ethical guidelines and approved by the Institutional Animal Care and Use Committee (IACUC) under license number 2019-006, during the daytime light cycle.

### 2.4. Molecular docking

The Molecular Operating Environment (MOE) v2019 (Chemical Computing Group, Montreal, QC, Canada) was used to the perform molecular docking studies with human protein A1Rs and ENT1, to evaluate the potential binding mechanism of LEV. The LEV structures were created in MOE using the Builder module and then optimized using the MMFF94x force field after being geometry corrected and protonated. X-ray crystal structures of A1Rs and ENT1, with resolutions of 3.30 and 2.90, were obtained from the Protein DataBank using accession codes 5N2S and 6OB6, respectively [22, 23]. The MOE Structure Correction module was used to add incomplete loops and rectify the configuration of the structures. The induce fit protocol was then employed to dock LEV into the binding site of the A1Rs and ENT1 using Triangular Matcher as the placement method and London dG and GBVI/WSA dG as the scoring and rescoring functions, respectively. Chimera software (Resources for Biocomputing, Visualization and Informatics, San Francisco, CA USA) was used for the visualization and graphics [24].

### 2.5. Acute PTZ-induced seizure model

**Figure 1:**
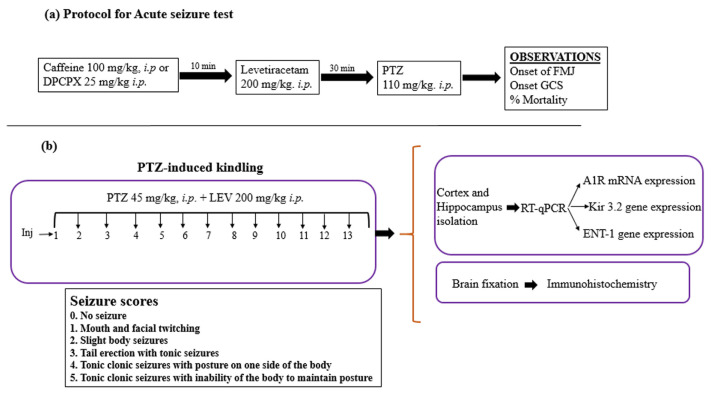
Protocols for the acute and kindling models.

The mice were placed in the testing room 1 h before the experiment. Details of the groups and treatments are given in Table 2. Adenosine receptor blockers (caffeine at 100 mg/kg or DPCPX at 25 mg/kg) were given 10 min before administering the LEV (200 mg/kg). PTZ at 110 mg/kg was injected intraperitoneally (i.p.) 30 min after LEV administration. After administering the PTZ, the onset of the first myoclonic jerk (FMJ), generalized clonic seizures (GCSs), and percent mortality were recorded. Figure 1a depicts the study protocol.

### 2.6. PTZ-induced kindling model of epileptogenesis

This study employed a total of 40 mice. Table 3 shows the distribution of the mice into 5 groups (n = 8 per group). Figure 1b depicts the study protocol. On every alternate day, the seizure scores were recorded for 30 min following the administration of a subconvulsive dose of PTZ (45 mg/kg, i.p.). The Racine scale [25] was used to assign scores: 1 (mouth and facial twitching), 2 (slight body jerks with head nodding), 3 (clonus with tail erection), 4 (tonic-clonic seizure with posture on one side of the body), and 5 (tonic-clonic seizure with loss of righting reflex). When 3 consecutive occasions of a score of 4/5 appeared in the PTZ group, the mice were considered kindled. To kindle all of the mice in the PTZ group, a total of 13 injections were needed. The mice were anaesthetized 24 h after the kindling was completed by administering a ketamine/xylazine cocktail. Cardiac perfusion was carried out using 1× phosphate buffer saline (PBS). The brain samples were cautiously removed; the hippocampus and cortex were isolated and processed for total RNA extraction using the TRIzol method.

### 2.7. Complementary DNA synthesis

A cDNA synthesis kit was used to create complementary DNA from isolated RNA samples (Invitrogen, cDNA Synthesis kit 1622; Thermo Fisher Scientific Inc., Waltham, MA, USA). Briefly, 1 μg of total RNA, 1 μL of 10X reaction buffer with MgCl2, and 1 μg of DNase-1 were mixed, and the final volume was brought to 10 μL with nuclease-free water. The mixture was incubated at 37 °C in a water bath for 30 min. After incubation, 1 μL of 50 mM EDTA was added to the tube and reincubated at 65 °C for 5 min. To this mixture, I μL of Oligo dT18 was added, followed by the addition of 4 μL of 5X reaction buffer, 1 μL of Ribolock RNase inhibitor, 2 μL of the dNTP mixture, and 1 μL of reverse transcriptase. It was incubated for 60 min at 42 °C followed by 70 °C for 5 min.

### 2.8. Reverse transcription-quantitative polymerase chain reaction (RT-qPCR) reaction

RT-qPCR reactions were prepared in low-profile PCR tubes. For amplification of the cDNA, SYBR Green PCR Master Mix (Themo Fisher Scientific, Oxford, UK) was used according to the manufacturer’s instructions. The protocol for the reactions was set at 95 °C for 10 min followed by 40 cycles of 95 °C for 15 s (denaturation), 60 °C for 15 s (annealing), and 72 °C for 30 s (extension) and melting curve analysis between 65 °C to 95 °C (at 5 s increments). After completing the PCR, the data were exported to an excel file and analyzed in Origin 8.5 statistical software (OriginLab Corp., Northampton, MA, USA).

### 2.9. Antibodies

Polyclonal antiA1R antibody (ADORA1) was used as a primary antibody while goat antirabbit antibody was used as a fluorescent secondary antibody. This primary antibody was used at a dilution of 1:400 for immunohistochemistry. Cy3-IgG goat antirabbit (secondary antibody) was used at a dilution of 1:500. For the nuclear staining, 4′,6-diamidino-2-phenylindole (DAPI) was used.

### 2.10. Immunofluorescence

The mice were anesthetized using ketamine/xylazine at doses of 90 and 10 mg/kg, respectively, followed by cardiac perfusions with heparinized PBS and 10% neutral buffer formalin. The brains of each animal were isolated and postfixed in 10% neutral buffer formalin (NBF) for 24 h. The next day, the brain tissues were immersed in 30% sucrose solution for 24 h, until the samples sank to the bottom of the jar. The brain tissues were embedded in optimum cutting temperature (OCT) media and kept at −20 °C. A SLEE cryotome (30 μm) was used to cut slices of the embedded brain tissues.

For the immunohistochemistry, the samples were incubated for 60 min at 37 °C with the blocking agent (RotiBlock solution). They were then incubated for 2 h at 37 °C with primary antibody (ADORA1 goat antirabbit, 1:400 dilution), followed by 1 h at 37 °C with secondary fluorescent antibody (Cy3-goat antirabbit antibody, 1:500 dilution). Next, the samples were rinsed and stained with DAPI. Fluoromount was used to mount the slides. The next day, fluorescent images were taken with an Eclipse NI-E Microscope (Nikon Corp., Minato City, Tokyo, Japan) and fluorescence intensity was assessed using ImageJ software (Wayne Rasband, NIH, Bethesda, USA).

### 2.11. Statistical analysis

The protein quantification density of the micrograph of the immunohistochemistry study was obtained using ImageJ software. The data were expressed as the mean ± standard error of the mean (SEM). The data were analyzed using 1-way analysis of variance (ANOVA) with the post hoc Tukey test using OriginLab v.8.5. *, **, and *** represented p < 0.05, p < 0.01, and p < 0.001, respectively.

## 3. Results

### 3.1. Molecular docking studies of the A1Rs and ENT1

**Figure 2:**
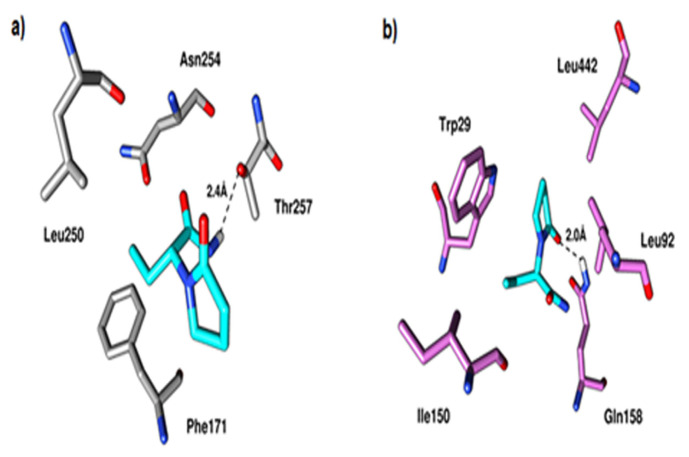
Binding mode of LEV at the binding site of (a) A_1_R (PDB ID 5N2S) and (b) ENT1 (PDB ID 6OB6). Protein residues are shown in grey for the A1Rs and orchid for the ENT1 as a stick, while the ligands are shown in different colors. Black dotted lines represent the hydrogen bond contacts.

The docking study found that LEV fit nicely into the orthosteric ligand binding pocket of the A1Rs, with binding affinity values of −5.68 and −5.43 kcal/mol, respectively. With LEV, the terminal amide nitrogen mediated a hydrogen bond with Thr257 at a distance of 2.4 (Figure 2a). Similarly, hydrophobic interactions with Phe171, Leu250, and Asn254 were observed.

Similarly, the molecular docking results showed that LEV bound to the orthosteric binding site of ENT1 with a binding affinity of −5.75 kcal/mol. The oxopyrrolidin ring of LEV was stacked with Trp29, and the oxygen in the oxo group mediated a hydrogen bond with Gln158 at a distance of 2.0 (Figure 2b). Similarly, amino acids Leu92, Ile150, and Leu442 were observed to facilitate hydrophobic interactions with the compound.

### 3.2. Caffeine and DPCPX attenuated the antiseizure activity of LEV in the acute PTZ-induced seizure model

**Figure 3:**
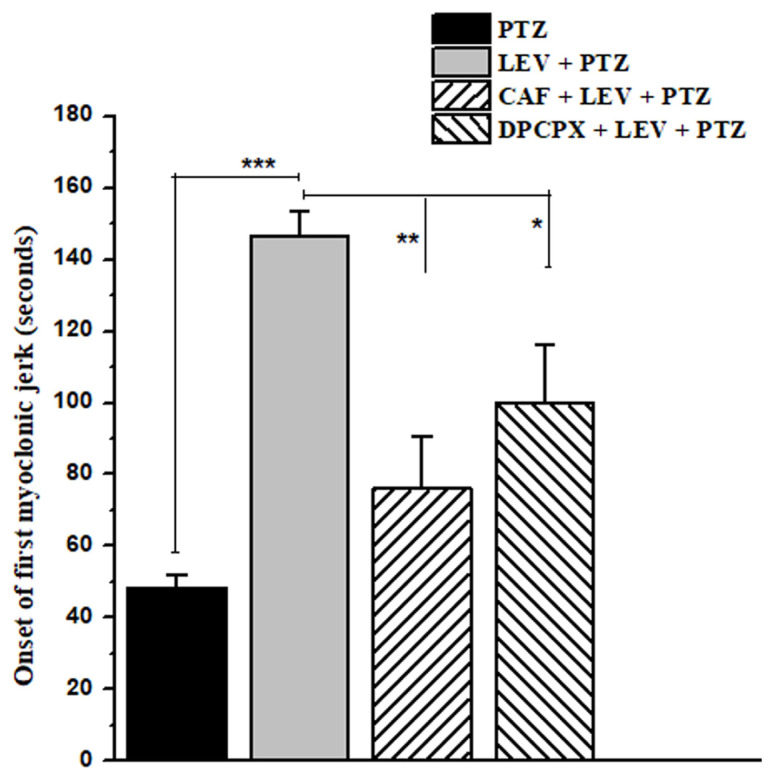
Caffeine and DPCPX attenuated the LEV-mediated delayed-onset of the FMJ in the acute PTZ-induced seizure model. Data are shown as the mean ± SEM, where n = 8, and *, **, and *** represent p < 0.05, p < 0.01, and p < 0.001, respectively.

Caffeine (100 mg/kg, i.p.) or DPCPX (25 mg/kg, i.p.) administration greatly decreased the antiseizure action of LEV (200 mg/kg, i.p.). With LEV, the FMJ appeared at 146.66 ± 6.76 s after PTZ administration, which was significantly (*F*_1, 14_ = 116.55, p < 0.001) delayed, particularly in comparison to the PTZ control group, where the FMJ occurred at 48.33 ± 3.75 s (Figure 3). Caffeine or DPCPX administration before LEV administration reversed the antiseizure activity of LEV by minimizing the delay of the FMJ to 76 ± 14.52 s (*F*_1, 14_ = 44.24, p < 0.01) and 100 ± 16.32 s (*F*_1, 14_ = 16.09, p < 0.05), respectively, when compared to LEV at 200 mg/kg (146.66 ± 6.76 s, Figure 3).

Furthermore, when compared to the PTZ control group, LEV (200 mg/kg, i.p.) followed by PTZ at 110 mg/kg, i.p. provided 100% protection. Caffeine and DPCPX were found to completely eliminate the % protection caused by LEV (Table 4). Similarly, PTZ administration caused the onset of a GCS at 66.66 ± 6.01 s. LEV, on the other hand, prevented the onset of GCSs in the mice given an acute convulsive dose of PTZ. As can be seen in Table 4, caffeine (100 mg/kg) and DPCPX (25 mg/kg) reversed the effects of LEV by producing GCSs in 115 ± 16.52 s and 142.33 ± 12.17 s, respectively.

### 3.3. Effect of LEV in the PTZ-induced kindling model

**Figure 4:**
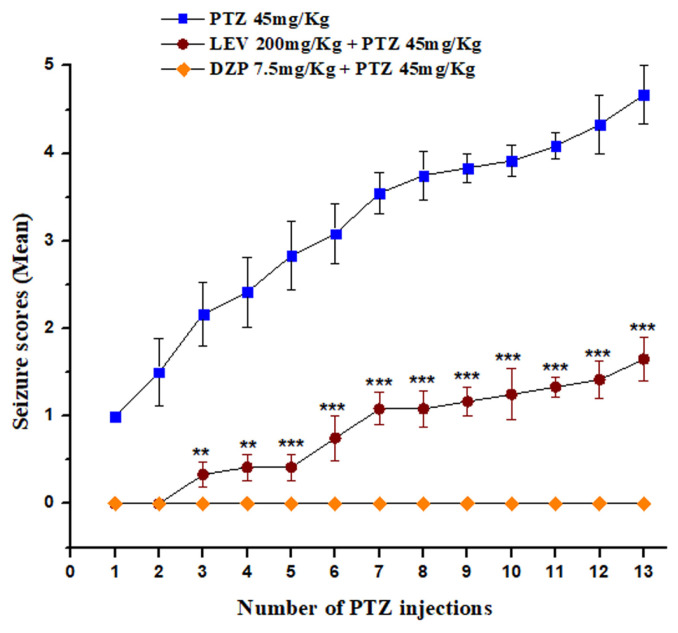
LEV significantly reduced the mean seizure score in the PTZ-induced kindling. Data were analyzed using 1-way ANOVA using Origin statistical software and expressed as the mean ± SEM. ***p < 0.001 was considered significant vs. the PTZ-treated group.

The repeated administration of a subconvulsive dose of PTZ (45 mg/kg, i.p.) on every alternate day gradually increased the seizure scores in the mice to 4/5 (clonic-tonic seizures) (Figure 4). Diazepam at a dose of 7.5 mg/kg significantly prevented seizure development in the mice. Furthermore, LEV (200 mg/kg, i.p.) significantly reduced the seizure score in the PTZ-induced kindled mice (p < 0.001, Figure 4). Remarkably, none of the mice received a score higher than 3, indicating the significant antiepileptogenic activity of the LEV.

### 3.4. Effect of LEV on the gene expression of A1Rs in the kindling model

**Figure 5:**
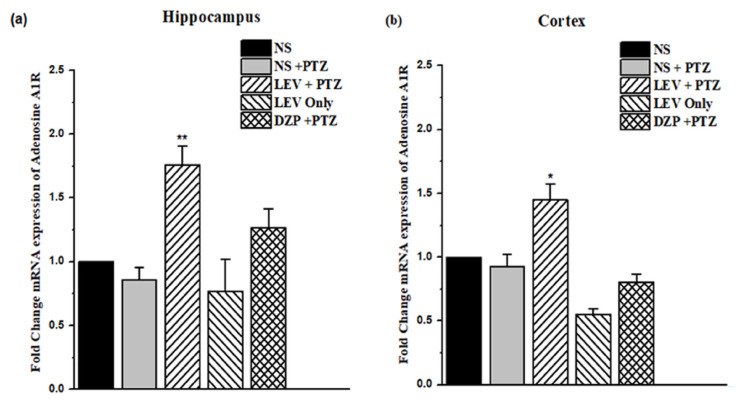
Changes in the gene expression of the A1Rs in the kindling model are shown in both (a) the hippocampus and (b) the cortex. Data were analyzed using 1-way ANOVA followed by the post hoc Tukey test and expressed as the mean ± SEM. *p < 0.05 and **p < 0.01 were considered significant when compared to the PTZ-treated group.

In the kindling model of epilepsy, LEV at 200 mg/kg increased the fold change mRNA expression in the A1Rs in the RT-qPCR by 1.76 ± 0.14 (*F*_1, 14_ = 46.73, p < 0.001) and 1.45 ± 0.12 (*F*_1, 14_ = 10.94, p < 0.05) times in the hippocampus and cortex, respectively, particularly in comparison with the PTZ-treated mice, i.e. G3 vs. G2 (Figure 5). Furthermore, LEV at 200 mg/kg (G4) reduced the fold change of the A1Rs in the hippocampus and cortex.

### 3.5. Gene expression of Kir3.2 in the brain of PTZ-kindled mice receiving treatment of LEV

**Figure 6:**
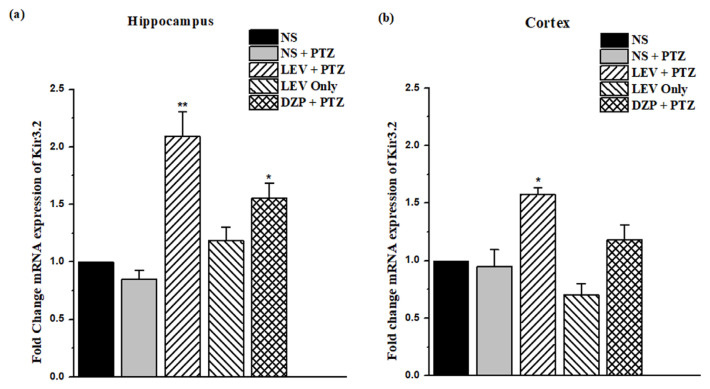
Gene expression of Kir3.2 in the kindled mice treated with LEV. (a) Kir3.2 gene expression changes in the hippocampus and (b) gene expression changes in the cortex following treatment with LEV (200 mg/kg). Data were analyzed using 1-way ANOVA followed by the post hoc Tukey test and expressed as the mean ± SEM. *p < 0.05 and **p < 0.01 were considered significant vs. the PTZ group.

Kir3.2 play a crucial part in the regulation of neuronal over-excitation. Figure 6 shows that LEV (200 mg/kg, i.p.) hindered PTZ-induced seizures in the kindling model by increasing Kir3.2 expression levels in the hippocampus (fold change: 2.09 ± 0.21, *F*_1, 14_ = 26.43, p > 0.01) and cortex (fold change: 1.57 ± 0.06, *F*_1, 14_ = 15.39, p < 0.05) when compared to the control group, i.e. G3 vs. G2 (Figure 6). In addition, it was found that diazepam at 7.5 mg/kg i.p. substantially increased Kir3.2 gene expression in the hippocampus (G5 vs. G2, *F*_1, 14_ = 10.45, p < 0.05).

### 3.6. Impact of LEV on the gene expression of ENT1 in the kindling model of epilepsy

**Figure 7:**
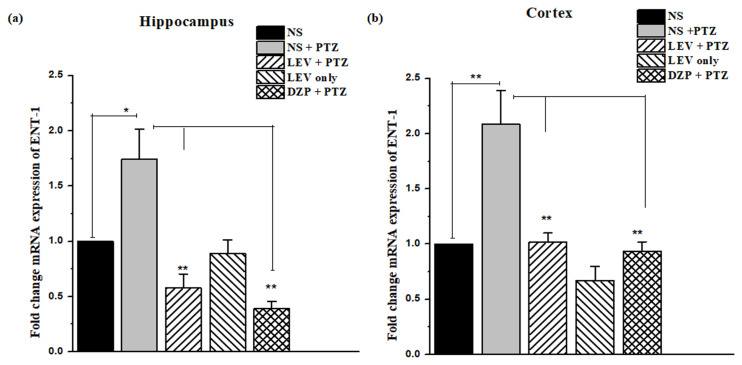
(a) The expression of ENT1 in the hippocampus and (b) gene expression changes in the cortex. Data were analyzed using 1-way ANOVA followed by the post hoc Tukey test and expressed as the mean ± SEM. ** and * denote p < 0.01 and p < 0.05, respectively.

ENT1 is important in the regulation of synaptic adenosine levels in the brain. The subconvulsive dose of PTZ (45 mg/kg, i.p.) increased the mRNA expression of ENT1 in the kindling model of epilepsy in the hippocampal (fold change: 1.74 ± 0.27, *F*_1, 14_ = 13.51, p < 0.05) and cortical regions (fold change: 2.09 ± 0.31, *F*_1, 14_ = 16.52, p < 0.01) in the RT-qPCR (Figure 7), when compared with the expression level of the control group (G1 vs. G2). In comparison to the PTZ group, LEV significantly reduced the fold change of the mRNA expression of ENT1 in the hippocampus (fold change: 0.58 ± 0.13, *F*_1, 14_ = 28.02, p < 0.01) and cortex (1.02 ± 0.08, *F*_1, 14_ = 21.7, p < 0.01, G2 vs. G3).

### 3.7. Effect of LEV on the immunofluorescence intensity of ADORA1 in the kindling model of epilepsy

**Figure 8:**
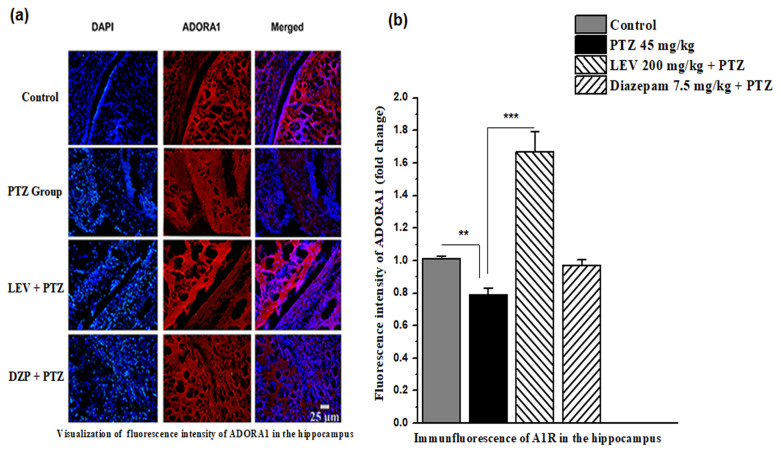
Immunofluorescent images of the ADORA1 expression in the hippocampus. (a) Intensity of the immunofluorescence of the A1Rs in the hippocampus in kindling model of epilepsy and (b) the fold increase in intensity of ADORA1 in the hippocampus. Data in 8b were analyzed using 1-way ANOVA followed by the post hoc Tukey test and expressed as the mean ± SEM. ** and *** denote p < 0.01 and p < 0.001, respectively.

**Figure 9:**
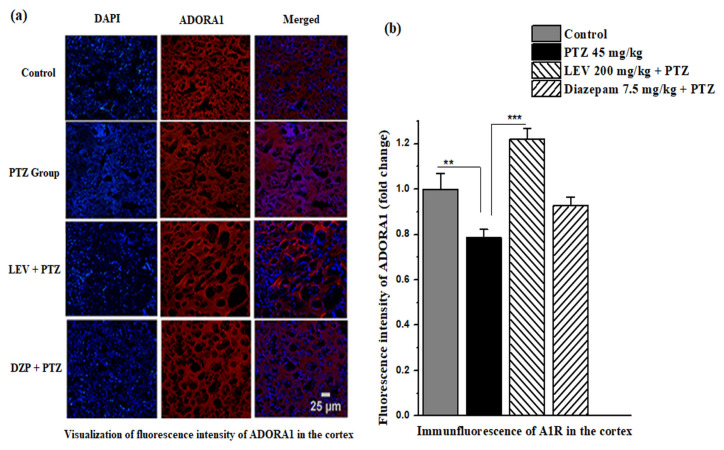
Photomicrographs of the ADORA1 expression in the cortex. (a) Intensity of the immunofluorescence of the A1Rs in the cortex in the kindling model of epilepsy and (b) the fold increase in intensity of ADORA1 in the cortex. Data were analyzed using 1-way ANOVA followed by the post hoc Tukey test and expressed as the mean ± SEM. ** and *** denote p < 0.01 and p < 0.001, respectively.

In the PTZ-induced kindling model of epilepsy in mice, the immunohistochemical analysis showed that LEV increased the fluorescence intensity of the A1R expression in the hippocampus (Figures 8a–8b) and cortex (Figures 9a–9b). Figure 8a shows that PTZ decreased the A1 receptor expression in the hippocampus to 0.79 ± 0.04 (*F*_1, 14_ = 25.02, p < 0.01). when compared to the control group. Furthermore, LEV substantially increased the A1R expression by 1.67 ± 0.12-fold when compared to the expression level of the control group (*F*_1, 14_ = 45.40, p < 0.001).

Similarly, in the cortex, the protein expression of the A1Rs decreased significantly (*F*_1, 14_ = 10.65, p < 0.01) in the PTZ treated group to 0.79 ± 0.04-fold when compared to the control group, as shown in Figure 9a. LEV increased the A1R expression in the cortex by 1.22 ± 0.05-fold when compared to the PTZ-treated group in the kindling model of epilepsy in mice (*F*_1, 14_ = 56.12, p < 0.001, Figure 9a).

## 4. Discussion

Conventional AEDs have multiple side effects. Therefore, attempts are being carried out to develop new drugs with minimal side effects and a broader spectrum of efficacy in epileptic patients. Among the novel AEDs, LEV has been regarded as the safest in terms of an adverse effect profile [8]. LEV has also been demonstrated to greatly reduce seizures in the kindling model of epilepsy and partially protect mice against seizures in acute seizure models [26]. Previous studies have reported that LEV acts peripherally on A1Rs to produce antihyperalgesic activity, which is counteracted by adenosine receptor antagonists, i.e. caffeine (nonselective antagonist) and DPCPX (selective A1R antagonist). The caffeine dose was chosen based on a prior investigation which demonstrated that a caffeine dose of 100 mg/kg had no interaction with PTZ in an animal model, whereas lower doses of 5 or 50 mg/kg had proconvulsant effects. This is because caffeine inhibits A1Rs and hence, has proconvulsant properties at low doses, whereas at larger doses, caffeine has strong effects on A2R and thus, neutralizes the effects of caffeine on A1Rs [15, 28]. The actions of LEV on the brain adenosinergic network have not been adequately investigated. Hence, antiepileptic effects of LEV were examined herein by modifying the adenosinergic pathway.

A1Rs hyperpolarize postsynaptic terminals, thus decreasing the release of neurotransmitters from nerve terminals. The hyperpolarizing action of A1Rs is associated with the activation of G-protein-coupled receptor-linked Kir3.2. These channels are also activated by G-protein-coupled receptors for gamma-aminobutyric acid (GABA_B_) receptors. However, GABA-mediated hyperpolarization via Kir3.2 was found to be less significant than that mediated by A1R activation. Additionally, the presence of adenosine was crucial for effective Kir3.2 effects [21].

The present study was divided into 3 sections: molecular docking investigations, an in vivo acute seizure test, and a chronic kindling model study. The docking experiments revealed that LEV interacts strongly with A1R and ENT1 binding sites. Caffeine and DPCPX were observed to decrease the antiepileptic activity of LEV in the acute seizure test, showing that LEV may moderately function on the adenosinergic pathway to reduce seizures. To validate these findings, changes in the A1Rs and its associated Kir3.2, as well as ENT1 gene expression, were investigated in the kindling model of epileptogenesis. In the kindling, LEV increased the A1Rs and their related Kir3.2 mRNA expression, while decreasing the gene expression of ENT1 in the hippocampus and cortex. Moreover, in the immunohistochemical analysis, it was also observed that LEV increased the protein expression of the A1Rs.

In human temporal lobe epilepsy, the density of the A1Rs is increased [29] or decreased in the temporal neocortical region of the brain [21]. In animal models, however, the A1R density is decreased in rat hippocampal pyramidal neurons [30]. Status epilepticus has been observed in A1R knockout mice following brain injury [31]. Herein, the density of the A1Rs was reduced in the cortical and hippocampal brain sections excised from the PTZ-treated rats. Nonetheless, it is hypothesized that LEV treatment increased the gene expression of the A1Rs in the brain of the mice, resulting in a reduction in seizures due to the inhibitory tone on neurons generated by A1R activation. Knocking down Kir3.2 in rats has previously been shown to increase seizure susceptibility [21, 32]. GABA_B_ receptor-mediated hyperpolarization was formerly thought to be caused by Kir3.2 activation. Studies have revealed that LEV has no effect on GABA receptors [33], and the activity of LEV mediated action on the Kir3.2 is connected to A1R activation. It is worth mentioning that diazepam increased the Kir3.2 gene expression in the hippocampus, but the exact mechanism of this increase is unknown.

The synaptic adenosine level in the brain is regulated by ENTs. The major transporter for adenosine regulation in the brain is ENT1 [34]. It has been observed that the ENT1 expression increases in various pathophysiological circumstances, including epilepsy and several inflammatory brain illnesses [35]. It was also revealed that the ENT1 expression increased in epileptic humans and animal models of epilepsy, and that pharmacological inhibition of ENT1 transporters in epileptic animal models decreased the seizure intensity [36]. The current study found that LEV lowered the over-expression of ENT1 mRNA in the kindling model of epilepsy. Seizure-induced astrogliosis may promote an increase in ENT1 expression, and LEV lowered the ENT1 gene expression in an animal model by suppressing astrogliosis [35]. It can be suggested that LEV prevents the hallmark feature of epilepsy, i.e. astrogliosis.

### 5. Limitations

The goal of epilepsy treatment is to achieve seizure freedom with minimal or no adverse events. For a better understating of the effects of LEV on the adenosine system, the determination of the adenosine levels in the brain tissues can strengthen the hypothesis.

In next step, we will evaluate the treatment in female mice to determine the differential effects in both sexes. Since adenosine has prominent effects on the heart, the side effects associated with LEV on the cardiac tissue will also be evaluated.

## 5. Conclusion

Based on the current docking studies and experimental data, it is possible to conclude that LEV enhances A1R and Kir3.2 mRNA expression in both the hippocampal and cortical regions of the brain, lowering seizure scores. Decreased ENT1 gene expression was observed, which suggests that LEV may increase the extracellular amount of adenosine. The immunohistochemistry examination revealed enhanced immunofluorescence intensity of ADORA1 in the brain, confirming the expression of A1Rs. The antiepileptogenic effects of LEV are thought to be caused by an increase in A1Rs and a decrease in ENT1 via the adenosinergic pathway.

## Figures and Tables

**Table 1:** Sequences of the primers used.

S. No1	Primer	Forward and reverse strand sequence	Product size
1	GAPDH	(F) AACTTTGGCATTGTGGAAGG (R) ACACATTGGGGGTAGGAACA	223
2	Adenosine A1R	(F) GCCCGGAAATGTACTGGTGA (R) GGCAGGTGTGGAAGTAGGTC	170
3	ENT1	(F) AGCCAGACAGGGCTCGATA (R) GTGACTGGTTGTCATGGCTC	103
4	Kir3.2 (GIRK2)	(F) GACAAACCCAGCATGCACAA (R) TTAGAGGGCCAGCAGTCAAG	198

GAPDH: glyceraldehyde 3-phosphate dehydrogenase, ENT: equilibrative nucleoside transporter, Kir3.2: potassium inwardly-rectifying channel 3.2.

**Table 2:** Treatment groups for the acute PTZ-induced seizure model.

Groups	Treatment (i.p.)
Group 1	PTZ (110 mg/kg)
Group 2	LEV (200 mg/kg) followed by f (PTZ 110 mg/kg) after 30 min.
Group 3	Caffeine (100 mg/kg) followed by LEV 200 mg/kg and then after 30 min administration of PTZ (110 mg/kg) was done.
Group 4	DPCPX (25 mg/kg) followed by LEV and then after 30 min PTZ (110 mg/kg) was administered.

PTZ: pentylenetetrazole, LEV: levetiracetam, DPCPX: 8-cyclopentyl-1,3-dipropylxanthine, i.p.: intraperitoneally.

**Table 3:** Treatment groups of the PTZ-induced kindling model of epilepsy.

Groups	Treatment (i.p.)
Group 1 (G1)	Saline control group (only normal saline was administered to these mice).
Group 2 (G2)	PTZ (45 mg/kg every alternate day).
Group 3 (G3)	LEV 200 mg/kg + PTZ (45 mg/kg every alternate day).
Group 4 (G4)	LEV (200 mg/kg) only.
Group 5 (G5)	Diazepam 7.5 mg/kg + PTZ (45 mg/kg every alternate day).

**Table 4:** GCSs and % mortality in the acute PTZ-induced seizure model.

Treatment	Onset time of the GCSs (s)	% Mortality
PTZ 110 mg/kg	66.66 ± 6.009	100%
LEV 200 mg/kg + PTZ 110 mg/kg	No GCSs	0 (No mortality)
CAF 100 mg/kg +LEV 200 mg/kg + PTZ 110 mg/kg	115 ± 16.52	100%
DPCPX 25 mg/kg + LEV 200 mg/kg + PTZ 110 mg/kg	142.33 ± 12.170	100%

Data are represented as the mean ± SEM of n = 8 mice/group. GCSs: Generalized clonic seizures, CAF: caffeine.

## References

[1] Akyuz E, Koklu B, Uner A, Angelopoulou E, Paudel YN (2022). Envisioning the role of inwardly rectifying potassium (Kir) channel in epilepsy. Journal of Neuroscience Research.

[2] Anderson WW (2020). Epileptogenesis Cortical Plasticity.

[3] Angelatou F, Pagonopoulou O, Maraziotis T, Olivier A, Villemeure JG (1993). Upregulation of A1 adenosine receptors in human temporal lobe epilepsy: a quantitative autoradiographic study. Neuroscience Letters.

[4] Ardid D, Lamberty Y, Alloui A, Coudore-Civiale MA (2003). Antihyperalgesic effect of levetiracetam in neuropathic pain models in rats. European Journal of Pharmacology.

[5] Arican P, Gencpinar P, Cavusoglu D, Dundar NO (2018). Levetiracetam monotherapy for the treatment of infants with epilepsy. Seizure.

[6] Boison D, Borea P, Varani K, Gessi S, Merighi S, Vincenzi F (2018). Regulation of Extracellular Adenosine. The Adenosine Receptors The Receptors.

[7] Boison D (2007). Adenosine as a modulator of brain activity. Drug News Perspective.

[8] Borea PA, Gessi S, Merighi S, Vincenzi F, Varani K (2018). Pharmacology of adenosine receptors: the state of the art. Physiological Reviews.

[9] Briggs DE, French JA (2004). Levetiracetam safety profiles and tolerability in epilepsy patients. Expert Opinion and Drug Safety.

[10] Chen YH, Kuo TT, Huang EYK, Hoffer BJ, Chou YC (2018). Profound deficits in hippocampal synaptic plasticity after traumatic brain injury and seizure is ameliorated by prophylactic levetiracetam. Oncotarget.

[11] Cheng RK, Segala E, Robertson N, Deflorian F, Doré AS (2017). Structures of human A1 and A2A adenosine receptors with xanthines reveal determinants of selectivity. Structure.

[12] De Smedt T, Raedt R, Vonck K, Boon P (2007). Levetiracetam: the profile of a novel anticonvulsant drug—part I: preclinical data. CNS Drug Reviews.

[13] Del Bianco C, Placidi F, Liguori C, Mari L, Ulivi M (2019). Long-term efficacy and safety of lacosamide and levetiracetam monotherapy in elderly patients with focal epilepsy: A retrospective study. Epilepsy & Behavior.

[14] Devinsky O, Vezzani A, Najjar S, De Lanerolle NC, Rogawski MA (2013). Glia and epilepsy: excitability and inflammation. Trends in Neuroscience.

[15] Esmaili Z, Heydari A (2019). Effect of acute caffeine administration on PTZ-induced seizure threshold in mice: Involvement of adenosine receptors and NO-cGMP signaling pathway. Epilepsy Research.

[16] Fredholm BB, Chen JF, Cunha RA, Svenningsson P, Vaugeois JM (2005). Adenosine and brain function. International Review in Neurobiology.

[17] Goddard TD, Huang CC, Ferrin TE (2005). Software extensions to UCSF chimera for interactive visualization of large molecular assemblies. Structure.

[18] Gouder N, Fritschy JM, Boison D (2003). Seizure suppression by adenosine A1 receptor activation in a mouse model of pharmacoresistant epilepsy. Epilepsia.

[19] Herman ST (2002). Epilepsy after brain insult: targeting epileptogenesis. Journal of Neurology.

[20] Ho SY, Chen IC, Chang KC, Lin HR, Tsai CW (2020). Equilibrative nucleoside transporters-1 inhibitors act as anti-epileptic agents by inhibiting glutamatergic transmission. Frontier in Neurosciences.

[21] Klitgaard H (2001). Levetiracetam: the preclinical profile of a new class of antiepileptic drugs?. Epilepsia.

[22] Kosmowska B, Ossowska K, Konieczny J, Lenda T, Berghauzen-Maciejewska K (2020). Inhibition of excessive glutamatergic transmission in the ventral thalamic nuclei by a selective A1R agonist, 5′-chloro-5′-deoxy-(±)-ENBA underlies its tremorolytic effect in the harmaline-induced model of essential tremor. Journal of Neuroscience.

[23] Löscher W, Brandt C (2010). Prevention or modification of epileptogenesis after brain insults: experimental approaches and translational research. Pharmacological Reviews.

[24] Micov A, Tomić M, Popović B, Stepanović-Petrović R (2010). The antihyperalgesic effect of levetiracetam in an inflammatory model of pain in rats: mechanism of action. British Journal of Pharmacology.

[25] Pitkänen A, Lukasiuk K (2011). Mechanisms of epileptogenesis and potential treatment targets. The Lancet Neurology.

[26] Racine RJ (1972). Modification of seizure activity by electrical stimulation: II. Motor seizure. Electroencephalography and Clinical Neurophysiology.

[27] Rebola N, Coelho JE, Costenla AR, Lopes LV, Parada A (2003). Decrease of adenosine A1 receptor density and of adenosine neuromodulation in the hippocampus of kindled rats. European Journal of Neuroscience.

[28] Signorini S, Liao YJ, Duncan SA, Jan LY, Stoffel M (1997). Normal cerebellar development but susceptibility to seizures in mice lacking G protein-coupled, inwardly rectifying K+ channel GIRK2. Proceedings of National Academy of Sciences.

[29] Spanoghe J, Larsen LE, Craey E, Manzella S, Van Dycke A (2021). The signaling pathways involved in the anticonvulsive effects of the adenosine A1 receptor. International Journal of Molecular Sciences.

[30] Specchio N, Wirrell EC, Scheffer IE, Nabbout R, Riney K (2022). International League Against Epilepsy classification and definition of epilepsy syndromes with onset in childhood: Position paper by the ILAE Task Force on Nosology and Definitions. Epilepsia.

[31] Stepanovic-Petrovic RM, Micov AM, Tomic MA, Ugrešic ND (2012). The local peripheral antihyperalgesic effect of levetiracetam and its mechanism of action in an inflammatory pain model. Anesthesia and Analgesia.

[32] Tabrizi N, Zarvani A, Rezaei P, Cheraghmakani H, Alizadeh-Navaei R (2019). Levetiracetam in genetic generalized epilepsy: A prospective unblinded active-controlled trial. Epilepsy Research.

[33] Tescarollo FC, Rombo DM, DeLiberto LK, Fedele DE, Alharfoush E (2020). Role of adenosine in epilepsy and seizures. Journal of Caffeine and Adenosine Research.

[34] Thurman DJ, Beghi E, Begley CE, Berg AT, Buchhalter JR (2011). Standards for epidemiologic studies and surveillance of epilepsy. Epilepsia.

[35] Wong M (2009). The window of epileptogenesis: looking beyond the latent period. Epilepsy Currents.

[36] Wright NJ, Lee SY (2019). Structures of human ENT1 in complex with adenosine reuptake inhibitors. Natural Structure and Molecular Biology.

